# Association between recurrent breast cancer and phthalate exposure modified by hormone receptors and body mass index

**DOI:** 10.1038/s41598-022-06709-3

**Published:** 2022-02-21

**Authors:** Pei-Jing Yang, Ming-Feng Hou, Fu Ou-Yang, Tsung-Hua Hsieh, Yen-Jung Lee, Eing-Mei Tsai, Tsu-Nai Wang

**Affiliations:** 1 Department of Public Health, College of Health Science, Kaohsiung Medical University, No.100, Shin-Chuan 1st Road, Sanmin Dist., Kaohsiung, 80708 Taiwan, ROC; 2 Division of Breast Oncology and Surgery, Department of Surgery, Kaohsiung Medical University Chung-Ho Memorial Hospital, Kaohsiung, 80756 Taiwan, ROC; 3 Department of Biomedical Science and Environmental Biology, College of Life Science, Kaohsiung Medical University, Kaohsiung, 80708 Taiwan, ROC; 4 Department of Medical Research, E-Da Hospital/E-Da Cancer Hospital, I-Shou University, Kaohsiung, 82445 Taiwan, ROC; 5 Center for Research Resources and Development, Kaohsiung Medical University, Kaohsiung, 80708 Taiwan, ROC; 6 Graduate Institute of Medicine, College of Medicine, Kaohsiung Medical University, No.100, Shin-Chuan 1st Road, Sanmin Dist., Kaohsiung, 80708 Taiwan, ROC; 7 Department of Obstetrics and Gynecology, Kaohsiung Medical University Chung-Ho Memorial Hospital, Kaohsiung, 80756 Taiwan, ROC; 8 Research Center for Environmental Medicine, Kaohsiung Medical University, Kaohsiung, 80708 Taiwan, ROC

**Keywords:** Breast cancer, Predictive markers, Breast cancer, Risk factors, Breast cancer, Cancer epidemiology

## Abstract

The association between phthalate exposure and breast cancer remains controversial. We performed a prospective patient cohort design to explore the interaction between creatinine-corrected urinary phthalate metabolites and hormone receptors as well as body mass index (BMI) on recurrent breast cancer. In this follow-up study, 636 female breast cancer patients and 45 new recurrent cases diagnosed for a total of 1576.68 person-years of follow-up were recruited. Mono-(2-ethyl-5-oxohexyl) phthalate (MEOHP) was negatively associated with breast cancer recurrence, with adjusted hazard ratio (aHR) 3rd vs. 1st quartile of 0.15 (95% CI 0.04–0.51). The MEOHP presented as a non-monotonic dose–response (NMDR) curve, being U-shaped. In the stratification of hormone receptors, MEOHP still exhibited a U-shaped dose–response curve. The third quartile of MEOHP showed significant lowest recurrent risk in the status of ER-positive (aHR 0.18, 95% CI 0.05–0.66), PR-negative (aHR 0.14, 95% CI 0.03–0.63), and HER2-negative (aHR 0.24, 95% CI 0.08–0.76). Whether in BMI < 25 or in BMI ≥ 25, the third quartile of MEOHP was negatively associated with recurrent breast cancer, and there was a negative interaction on an additive scale between MEOHP and BMI (*p*_interaction_ = 0.042). The association between MEOHP and recurrent breast cancer was modified by hormone receptors and BMI.

## Introduction

About 2.3 million breast cancer cases were newly diagnosed in 2020 worldwide. Breast cancer has surpassed lung cancer as the most common cancer, and the number of deaths was about 685,000. Breast cancer is the most commonly diagnosed cancer (24.5% of female cancers) and the leading cause of cancer death (15.5% of female cancer deaths) in women^1,2^. The highest estimated age-standardized incidence of breast cancer in most areas was between the ages of 60 and 74 years, which is available online at the International Agency for Research on Cancer (IARC) (https://gco.iarc.fr). In Taiwan, data from the Ministry of Health and Welfare (MOHW) showed that the age-adjusted incidence rates of breast cancer rise sharply to 45–49 years and are increasing over time; the age-adjusted incidence rates of 45–49 years in females were 64.96 per 100,000 in 2010 to 74.57 per 100,000 in 2015, and in 2018, it was 79.40 per 100,000. Furthermore, the 5-year survival rate of breast cancer between 2014 and 2019 was 88.6% in Taiwan. Studies have indicated that women with local recurrence have a higher risk of metastasis and death than women without local recurrence^3,4^.

Phthalates are man-made chemicals used in many products, including personal care products and consumer products^5^. Phthalates do not form a covalent bond with the plastic matrix and leak from containers to the surroundings, causing human exposure^6^, and as they are ubiquitous in air, dust, water, and food, humans could be exposed through inhalation, ingestion, and skin absorption^7,8^. Some artificial chemicals have similar structure to endogenous estrogen and can bind to estrogen receptors (ER)^9^. Phthalates are known environmental endocrine-disrupting chemicals (EDCs) that could bind to and interact with ER and progesterone receptor (PR)^10,11^; however, the association of phthalates with hormone-dependent cancer, such as breast cancer, remains conflicting.

A very recent prospective study indicated that phthalate metabolites in urine were not associated with breast cancer in postmenopausal women^12^. Three other retrospective case–control studies had inconsistent findings; one study suggested that most urinary phthalate metabolites were negatively associated with breast cancer and breast cancer-specific mortality among predominantly white women^13^; another Mexican study reported that mono-ethyl phthalate (MEP) was associated with breast cancer while mono-benzyl phthalate (MBzP) and mono-(3-carboxypropyl) phthalate (MCPP) were negatively related to breast cancer^14^, although the last study in Native Alaskans showed that mono-2-ethylhexyl phthalate (MEHP) was associated with breast cancer^15^. Other cancer epidemiological studies still had different findings. Thyroid cancer and benign nodule were associated with mono-methyl phthalate (MMP), mono-(2-ethyl-5-oxohexyl) phthalate (MEOHP), and MEHP, but mono-n-butyl phthalate (MnBP) was observed to have a negative correlation^16^. A population-based nested case–control study in Taiwan found that MBzP, MnBP, and mono-isobutyl phthalate (MiBP) were associated with prostate cancer in obese men^17^. So far, few epidemiological studies have addressed the relationship between phthalate exposure and recurrent breast cancer.

Phthalates are rapidly metabolized to the corresponding metabolites through hydrolysis and oxidation, making phthalates more soluble and easily excreted into the urine^18^. Urine is a non-invasive sample and easier to obtain than blood, and most phthalate metabolites are easily detectable in urine than in other body fluids^19^; furthermore, the concentration of phthalate metabolites in urine is about 50 times higher than in blood, so quantifying urinary phthalate metabolite concentrations is the best way to assess phthalate exposure in epidemiological studies^20,21^.

To our best knowledge, this is the first study using a prospective patient cohort design to explore the interaction between phthalate exposure and hormone receptors as well as body mass index (BMI) on breast cancer recurrence.

## Methods

### Study population

Seven hundred and twenty-six female patients diagnosed with breast cancer by breast surgeon specialists and histopathological sections were recruited from Kaohsiung Medical University (KMU) Chung-Ho Memorial Hospital (a medical center in southern Taiwan) from September 2013 to June 2018 and follow-up of these patients were continued until July 2019. Patients under 20 years of age, being non-citizens (n = 1), having benign breast disease (n = 11), suffering from any other cancer (n = 1), and with incomplete data (n = 11) were excluded. Due to 66 patients not having urine samples, only 636 breast cancer patients completed phthalate metabolite examinations in urine.

**Figure 1:**
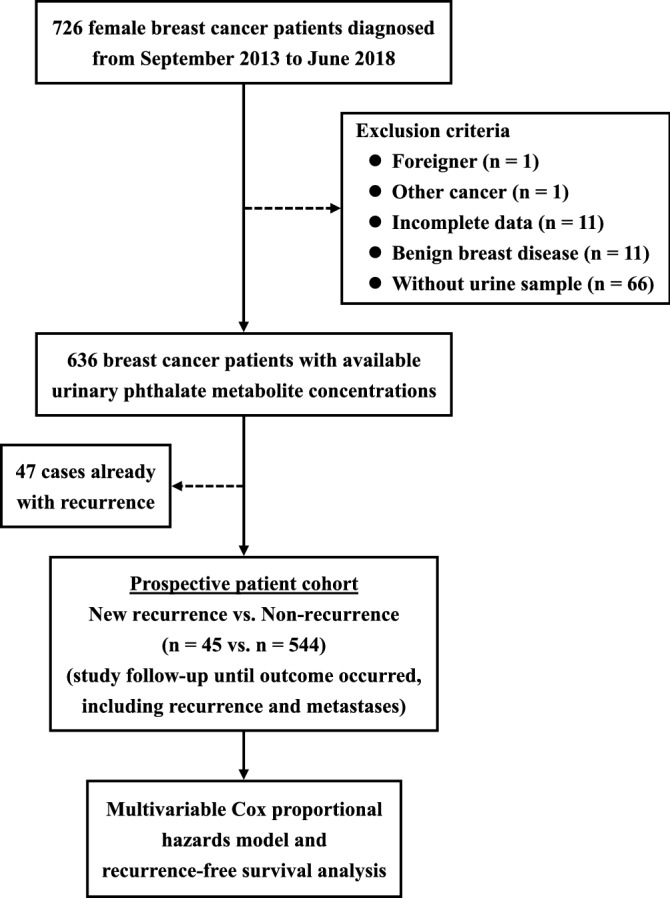
Study design and criteria for recruiting breast cancer patients.

In this patient cohort study, 636 breast cancer patients were recruited in this study and 47 patients already with recurrence were excluded in this study design and the patients were followed up every 6 months until the end of this study. For recurrence-free survival analyses, the breast cancer recurrence and metastases were study outcomes (recurrent patients including patients who died of breast cancer), while non-recurrent breast cancer patients were censored at the time of lost follow-up, withdrawal, and last follow-up date. Finally, a total of 589 non-recurrent breast cancer patients with 1576.68 person-years were followed up, and 45 new recurrent breast cancer cases were diagnosed. The prospective follow-up was performed to explore the association between phthalate exposure and the risk of breast cancer recurrence with new recurrent patients (n = 45) vs. non-recurrent patients (n = 544) (Fig. 1). Researchers regularly followed patients for breast cancer recurrence, metastasis, and survival every 6 months by medical record, and extracted the patient's medical history, including breast density, treatment, TNM stage (TNM classification of malignant tumors), ER, PR, human epidermal growth factor receptor 2 (HER2), etc. Immunohistochemistry (IHC) was used to test hormone receptors.

All these patients provided blood and spot morning urine samples and completed questionnaires with trained interviewers to obtain information concerning sociodemographic characteristics, alcohol use, smoking status, dietary habits, reproductive history, family history of breast cancer, etc. According to the standard classification of the World Health Organization (WHO), BMI was categorized as non-obesity (BMI < 25 kg/m^2^) and overweight or obesity (BMI ≥ 25 kg/m^2^). Written informed consents were obtained from all participants before data and biological specimen collection. The study protocol was approved by the Institutional Review Board (IRB) of Kaohsiung Medical University (KMU) Chung-Ho Memorial Hospital (IRB no. KMUHIRB-20120104, KMUHIRB-20140055, and KMUHIRB-G(I)-20150026). The study was performed in accordance with the Declaration of Helsinki.

### Phthalate reagents

Nine phthalate analytical standards were purchased from Cambridge Isotope Laboratories (CIL) (Andover, Massachusetts, USA); namely, MEP (Product Code: ULM-4585-MT-1.2), MnBP (ULM-6148-MT-1.2), MiBP (ULM-7919-MT-1.2), MEHP (ULM-4583-MT-1.2), mono-(2-ethyl-5-hydroxyhexyl) phthalate (MEHHP) (ULM-4662-MT-1.2), mono-(2-ethyl-5-carboxypentyl) phthalate (MECPP) (ULM-8149-MT-1.2), MEOHP (ULM-4663-MT-1.2), MMP (ULM-6697-MT-1.2), and MBzP (ULM-6149-MT-1.2). Nine phthalate internal standards were purchased from Toronto Research Chemicals (TRC) (North York, Ontario, Canada) and Cambridge Isotope Laboratories (CIL) respectively; namely, MEP-d4 (Cat. No.: M542582), MnBP-d4 (M525102), MiBP-d4 (M547702), MEHP-d4 (M542492), MEHHP-d4 (M542512), MECPP-^13^C4 (Product Code: CLM-8148-MT-1.2), MEOHP-^13^C4 (CLM-6640-MT-1.2), MMP-d4 (M566542), and MBzP-d4 (M524902).

### Sample preparation

The first spot morning urine samples were collected in a sterile brown glass bottle from all breast cancer participants and then frozen at − 20 °C until analysis. For analysis, urine samples were thawed at 4 °C, and then 1 mL urine sample was transferred to a glass tube, followed by the addition of 10 μL mixed isotope internal standards solution and 250 μL ammonium acetate (1 M, pH 6.5). After adding 3 μL beta-glucuronidase from *E. coli* K12 (Roche Diagnostics GmbH, Mannheim, Germany), the mixture was shaken and incubated for 90 min at 37 °C in a water bath, and then placed for 10 min at room temperature. The mixture was mixed with 2 mL phosphate buffer (pH 2) for 30 s and centrifuged at 3500 rpm for 10 min at 4 °C. The supernatant was purified with solid-phase extraction cartridge (Agilent ABS Elut-NEXUS) (Agilent Technologies, Palo Alto, CA, USA). After sample loading, 2 mL formic acid and 1 mL water were added to the cartridge, and then eluted with 2 mL acetonitrile and 2 mL ethyl acetate and dried by nitrogen gas at 55 ℃. The residue was reconstituted by adding 200 μL water and taken for liquid chromatography–tandem mass spectrometry (LC–MS/MS) analysis.

### Urinary phthalate metabolites assessment by LC–MS/MS

The concentrations of nine urinary phthalate metabolites: MEP, MnBP, MiBP, MEHP, MEHHP, MECPP, MEOHP, MMP, and MBzP, were measured by the same technician in double-blinded fashion using LC–MS/MS, which was a Waters ACQUITY UPLC system (Waters Corporation, Milford, MA, USA) coupled with a Finnigan TSQ Quantum Ultra triple-quadrupole MS (Thermo Fisher Scientific, San Jose, CA, USA) equipped with an electrospray ionization (ESI) source in a negative ion mode and combined with Xcalibur software (Thermo Fisher Scientific).

A 10 μL quantity of each sample was injected into a ACQUITY UPLC CSH Phenyl-Hexyl Column (2.1 × 100 mm, 1.7 μm) (Waters Corporation, Milford, MA, USA) equipped with a BEH C18 column VanGuard Pre-column (2.1 × 5 mm, 1.7 μm) (Waters Corporation) with a flow rate of 250 μL/min at 40 ℃ in the gradient from 30% solvent B (0.1% acetic acid in acetonitrile) for 0.0–5.0 min, then increased to 40% B for 10.0 min, 50% B for 12.0 min, and 100% B for 13.0 min, then reduced back down to 10% B in 13.1–15.0 min (solvent A: 0.1% acetic acid in water). The negative ion mode was set as follows: ion spray voltage, 3000 V; sheath gas (N_2_), 28 psi; auxiliary gas (N_2_), 10 psi; and collision gas (Ar), 1.0 mTorr. The detection rates, precursor ion (m/z), product ion (m/z), retention times (min), and collision energy (V) were shown in Supplementary Table S1.

### Method validation and sample determination

All experiments were done in the laboratory at the Center for Research Resources and Development in KMU, having an external quality assurance by German External Quality Assessment Scheme for toxicological analyses. Blank sample and quality control were simultaneously performed in each experiment along with the analytical protocol. The urinary concentrations of phthalate metabolites were adjusted by creatinine for urine dilution. Creatinine-adjusted units (μg/g) were calculated by dividing the phthalate metabolite concentrations (μg/L) by creatinine concentrations (mg/dl) and multiplying by 100^22^.

The Σ_4_DEHP was the sum of urinary MEHP, MEHHP, MECPP, and MEOHP concentrations to represent similar source exposure^23^. If the values of concentrations were less than the limit of detection (LOD), the values were replaced with LOD/$\sqrt 2$. The detection rates of MMP and MBzP were 0% and 68.9% separately; thus, the two metabolites were not considered in further analysis. The detection rates of the other seven urinary phthalate metabolite concentrations were 98.4% to 100%. We calculated the intra- and inter-batch coefficient of variation (CV) for individual metabolites. Intra-batch CV: MnBP (12.63%), MiBP (13.56%), MEHP (12.50%), MEHHP (7.89%), MECPP (13.85%), and MEOHP (9.97%). Inter-batch CV: MnBP (8.18%), MiBP (6.34%), MEHP (7.83%), MEHHP (11.19%), MECPP (11.76%), and MEOHP (4.47%).

### Statistical analysis

The continuous and categorical variables of sociodemographic and clinical characteristics for new recurrence and non-recurrence groups were compared using Independent sample *t*-test and Chi-square test or Fisher’s exact test. If the sociodemographic and clinical characteristics as risk factors associated with breast cancer were significantly different between the two groups, they were selected as potential confounding factors in the adjusted model. The values of creatinine-corrected urinary phthalate metabolite concentrations were non-normal distributions, so the Mann–Whitney U test or ln-transformed for Independent sample *t*-test were used to analyze the differences between two groups. Spearman correlation coefficients were used to evaluate correlations between individual phthalate metabolite concentrations. The concentrations were categorized into quartiles according to the distribution of phthalate metabolite concentrations in all participants with the first quartile being defined as the reference group.

Multivariable Cox proportional hazards model was performed for recurrence-free survival analysis with new recurrent breast cancer patients as related to phthalate exposure. The hazard ratios (HRs) and 95% confidence intervals (CIs) were used to estimate the risk of recurrent breast cancer in relation to phthalate exposure. The study patients were also stratified by the status of ER (ER-negative vs. ER-positive), PR (PR-negative vs. PR-positive), and HER2 (HER2-negative vs. HER2-positive) as well as BMI (BMI < 25 kg/m^2^ vs. BMI ≥ 25 kg/m^2^) to estimate the interaction effect of phthalate exposure and hormone receptors as well as BMI on the risk of breast cancer recurrence. The interaction was estimated for survival analysis based on additive scale in this prospective follow-up design^24,25^. The measurement for interaction effect as additive scale was relative excess risk due to interaction (RERI_HR_ = e^β1+β2+β3^ − e^β1^ − e^β2^ + 1)^24,26^.

Because the associations between recurrent breast cancer and eight phthalate metabolites were evaluated at the same time, we used Bonferroni correction (p-value multiplied by eight phthalate comparisons) to reduce the potential problems (Type I error) raised from multiple comparisons. The analyses were performed using SPSS 22.0 and all tests were two-sided. *p-*values < 0.05 were considered statistically significant.

### Ethics declarations

Written informed consents were obtained from all participants before data and biological specimen collection. The study protocol was approved by the Institutional Review Board (IRB) of Kaohsiung Medical University (KMU) Chung-Ho Memorial Hospital (IRB no. KMUHIRB-20120104, KMUHIRB-20140055, and KMUHIRB-G(I)-20150026). The study was performed in accordance with the Declaration of Helsinki.

### Consent to participate/consent to publish

Written informed consents were obtained from all participants before data and biological specimen collection. All the authors have read and approved the paper for publication.

## Results

### Patient characteristics in patient cohort

In Table 1, as of June 2018, 726 breast cancer patients were enrolled in this study, of which 636 patients underwent urinary phthalate metabolite examination. In this patient cohort study, 589 non-recurrent breast cancer were followed up with 1576.68 person-years, and 45 new recurrent breast cancer cases were diagnosed. Compared to non-recurrent patients, new recurrent patients were less married (70.5%), smoked more (15.9%), had higher breast cancer stages (59.1%), poorer cell differentiation (47.7%), larger tumor size (61.9%), less ER-positive status (66.7%), less PR-positive status (44.4%), more chemotherapy (88.9%), and more radiotherapy (80.0%).

**Table 1:** The sociodemographic and clinical characteristics of breast cancer patients.

Characteristics, n (%)	Full samples (n = 636)	New recurrence (n = 45)	Non-recurrence (n = 544)	*p-*values^a^
Age at diagnosis (year), mean ± SD	51.16 ± 10.46	52.29 ± 12.41	51.08 ± 10.29	0.457
BMI (kg/m^2^), mean ± SD	23.73 ± 3.86	23.53 ± 3.66	23.79 ± 3.90	0.667
Education (≥ college)	236 (37.8)	17 (38.6)	202 (37.6)	0.893
Marriage (married)	505 (80.9)	31 (70.5)	442 (82.5)	0.048*
Smoking state (ever)	27 (4.3)	7 (15.9)	19 (3.5)	0.002**
**Physical activity**				0.376
Never	274 (43.8)	19 (43.2)	238 (44.3)	
1–2 times a week	98 (15.7)	10 (22.7)	81 (15.1)	
3–6 times a week	253 (40.5)	15 (34.1)	218 (40.6)	
Age at menarche (≤ 13 years)	319 (51.1)	28 (63.6)	276 (51.5)	0.121
Menopause (premenopausal)	158 (25.3)	15 (34.1)	139 (25.9)	0.236
Oral contraceptive use (ever)	75 (12.0)	1 (2.3)	68 (12.7)	0.041*
Parity (parous)	528 (84.5)	35 (79.5)	453 (84.4)	0.403
Age at first birth (year), mean ± SD	26.96 ± 4.79	26.91 ± 4.61	27.04 ± 4.88	0.883
Lactation history (ever)	303 (57.4)	21 (60.0)	256 (56.5)	0.688
**Fried food**				0.742
1–2 times a week	589 (94.2)	41 (93.2)	505 (94.0)	
3–6 times a week	36 (5.8)	3 (6.8)	32 (6.0)	
Benign breast disease (Ever)	138 (22.1)	9 (20.5)	119 (22.2)	0.793
Family history of breast cancer (yes)	129 (20.6)	3 (6.8)	115 (21.4)	0.021*
**Breast density**				0.854
Fatty	7 (1.2)	0 (0.0)	6 (1.1)	
Mildly dense	84 (13.8)	5 (12.5)	67 (12.8)	
Moderately dense	428 (70.5)	28 (70.0)	378 (72.4)	
Extremely dense	88 (14.5)	7 (17.5)	71 (13.6)	
**Molecular subtype**				0.115
Luminal A	385 (63.4)	24 (53.3)	338 (65.5)	
Luminal B	103 (17.0)	6 (13.3)	83 (16.1)	
HER2-enriched	65 (10.7)	8 (17.8)	53 (10.3)	
Triple negative	54 (8.9)	7 (15.6)	42 (8.1)	
**TNM stage**				< 0.001**
0/I/II	521 (82.3)	18 (40.9)	483 (89.1)	
III/IV	112 (17.7)	26 (59.1)	59 (10.9)	
**Grade**				0.032*
Well differentiated	89 (14.8)	2 (4.5)	83 (16.1)	
Moderately differentiated	311 (51.7)	21 (47.7)	268 (52.1)	
Poorly differentiated	202 (33.6)	21 (47.7)	163 (31.7)	
Tumor size (> 2 cm)	261 (42.0)	26 (61.9)	203 (38.0)	0.002**
Invasive (yes)	477 (85.9)	38 (95.0)	401 (84.6)	0.074^#^
ER status (ER-positive)	499 (79.6)	30 (66.7)	432 (80.7)	0.024*
PR status (PR-positive)	422 (67.8)	20 (44.4)	374 (70.6)	< 0.001**
HER2 status (HER2-positive)	168 (27.6)	14 (31.1)	136 (26.3)	0.485
Chemotherapy (yes)	414 (65.1)	40 (88.9)	331 (60.8)	< 0.001**
Radiotherapy (yes)	378 (59.4)	36 (80.0)	313 (57.5)	0.003*
Hormone therapy (yes)	497 (78.1)	30 (66.7)	431 (79.2)	0.050^#^

*SD* standard deviation, *BMI* body mass index, *TNM stage* TNM classification of malignant tumors, *ER* estrogen receptor, *PR* progesterone receptor, *HER2* human epidermal growth factor receptor 2.

^a^*p-*values were calculated for continuous variables by Independent sample *t*-test and for categorical variables by Chi-square test or Fisher’s exact test.

^#^*p-*values < 0.1, *p-values < 0.05, **p-values < 0.01.

### Urinary phthalate metabolite profiles

Urinary phthalate metabolite concentrations were significantly positively related to each other as shown in Supplementary Table S2. The geometric mean concentrations of metabolites were not significant between the two groups (Supplementary Table S3). In the concentration of metabolites, the concentration of MEP was highest, followed by MECPP, and then MnBP.

### The results of prospective follow-up

The recurrence-free survival analysis of patient cohort was shown in Table 2. MEOHP were significantly negatively associated with breast cancer recurrence with adjusted HRs 3rd vs. 1st quartile of 0.15 (95% CI 0.04–0.51, *p*-value = 0.003). After performing Bonferroni correction for eight phthalate comparisons, only MEOHP was still significant (*p*-value = 0.024; 0.003 multiplied by eight phthalate comparisons). Furthermore, the dose–response curve of MEOHP presented as non-monotonic dose–response (NMDR), which was U-shaped.

**Table 2:** Association between recurrent breast cancer and urinary phthalate metabolite concentrations.

Metabolites^a^, n (%)	Prospective patient cohort follow-up			aHR (95% CI)^b^	*p*-values
	Person-years (n = 589)	New recurrence (n = 45)	Non-recurrence (n = 544)		
**MEP (μg/g)**					
0.74 to ≤ 9.94	356.71	11 (24.4)	137 (25.2)	1	
> 9.94 to ≤ 21.65	390.87	13 (28.9)	138 (25.4)	2.03 (0.80–5.15)	0.135
> 21.65 to ≤ 52.12	371.71	15 (33.3)	134 (24.6)	1.36 (0.57–3.26)	0.487
> 52.12 to 5303.46	457.38	6 (13.3)	135 (24.8)	0.47 (0.14–1.53)	0.208
**MnBP (μg/g)**					
1.63 to ≤ 10.51	317.76	14 (31.1)	136 (25.0)	1	
> 10.51 to ≤ 18.53	381.47	8 (17.8)	139 (25.6)	0.82 (0.30–2.28)	0.706
> 18.53 to ≤ 33.22	410.91	14 (31.1)	133 (24.4)	1.97 (0.84–4.62)	0.121
> 33.22 to 657.89	466.55	9 (20.0)	136 (25.0)	0.83 (0.34–2.05)	0.686
**MiBP (μg/g)**					
0.17 to ≤ 2.70	320.28	13 (28.9)	138 (25.4)	1	
> 2.70 to ≤ 5.69	345.15	13 (28.9)	140 (25.7)	0.79 (0.34–1.84)	0.583
> 5.69 to ≤ 16.56	368.54	13 (28.9)	123 (22.6)	1.02 (0.45–2.30)	0.963
> 16.56 to 1665.92	542.72	6 (13.3)	143 (26.3)	0.34 (0.11–1.10)	0.072^#^
**MEHP (μg/g)**					
0.28 to ≤ 2.91	299.81	16 (35.6)	138 (25.4)	1	
> 2.91 to ≤ 6.39	336.45	13 (28.9)	135 (24.8)	0.86 (0.38–1.94)	0.709
> 6.39 to ≤ 16.34	413.49	8 (17.8)	136 (25.0)	0.49 (0.20–1.20)	0.117
> 16.34 to 884.60	526.92	8 (17.8)	135 (24.8)	0.48 (0.17–1.30)	0.148
**MEHHP (μg/g)**					
0.80 to ≤ 7.43	410.45	10 (22.2)	132 (24.3)	1	
> 7.43 to ≤ 11.97	366.55	15 (33.3)	140 (25.7)	0.83 (0.35–2.01)	0.685
> 11.97 to ≤ 20.04	386.89	11 (24.4)	137 (25.2)	0.76 (0.29–2.01)	0.579
> 20.04 to 2831.20	421.80	9 (20.0)	135 (24.8)	0.81 (0.32–2.02)	0.644
**MECPP (μg/g)**					
0.83 to ≤ 12.94	360.84	10 (22.2)	137 (25.2)	1	
> 12.94 to ≤ 20.16	361.08	18 (40.0)	133 (24.4)	1.75 (0.73–4.21)	0.210
> 20.16 to ≤ 35.98	394.10	8 (17.8)	144 (26.5)	0.45 (0.15–1.40)	0.169
> 35.98 to 3971.76	460.66	9 (20.0)	130 (23.9)	0.93 (0.34–2.55)	0.888
**MEOHP (μg/g)**					
0.99 to ≤ 4.53	334.93	16 (35.6)	132 (24.3)	1	
> 4.53 to ≤ 7.23	357.24	13 (28.9)	133 (24.4)	0.58 (0.26–1.30)	0.188
> 7.23 to ≤ 11.82	405.58	5 (11.1)	148 (27.2)	0.15 (0.04–0.51)	0.003**^c^
> 11.82 to 2968.51	478.94	11 (24.4)	131 (24.1)	0.50 (0.22–1.18)	0.113
**Σ**_**4**_**DEHP (μg/g)**					
6.77 to ≤ 32.54	327.40	13 (28.9)	134 (24.6)	1	
> 32.54 to ≤ 50.69	354.48	17 (37.8)	134 (24.6)	1.52 (0.68–3.39)	0.309
> 50.69 to ≤ 86.16	418.39	3 (6.7)	145 (26.7)	0.09 (0.01–0.68)	0.020*^d^
> 86.16 to 10,656.06	476.42	12 (26.7)	131 (24.1)	0.79 (0.34–1.88)	0.600

*HR* hazard ratio, *CI* confidence interval.

^a^The urinary concentration unit of phthalate metabolites were μg/g creatinine and were categorized into quartiles with the first quartile being defined as the reference group.

^b^HR was calculated by multivariable Cox proportional hazards model and adjusted for marriage, smoking state, TNM stage, grade, tumor size, invasive, PR status, chemotherapy, and radiotherapy, shown as aHR (95% CI).

^c^The *p*-value was still significant after Bonferroni correction (*p*-value = 0.024; 0.003 multiplied by eight phthalate comparisons).

^d^The *p*-value was not significant after Bonferroni correction (*p*-value = 0.16; 0.02 multiplied by eight phthalate comparisons).

^#^*p-*values < 0.1, *p-values < 0.05, **p-values < 0.01.

### The interaction effect on breast cancer recurrence

The effect of MEOHP on the risk of breast cancer recurrence stratified by the status of ER, PR, and HER2 as well as BMI were illustrated as in Table 3. Compared to the first quartile, the third quartile of MEOHP presented the lowest breast cancer recurrence risk in ER-positive status (aHR 0.18, 95% CI 0.05–0.66, *p*-value = 0.009), PR-negative status (aHR 0.14, 95% CI 0.03–0.63, *p*-value = 0.011), and HER2-negative status (aHR 0.24, 95% CI 0.08–0.76, *p*-value = 0.015). The dose–response curves of MEOHP still presented as U-shaped (Supplementary Fig. S1). The association between MEOHP and recurrent breast cancer was significantly modified by BMI (*p*_interaction_ = 0.042). Compared to the first quartile, the third quartile of MEOHP presented the lowest breast cancer recurrence risk in BMI < 25 (aHR 0.25, 95% CI 0.07–0.93, *p*-value = 0.039) and MEOHP was still negatively associated with recurrent breast cancer in BMI ≥ 25 (aHR 0.11, 95% CI 0.01–0.92, *p*-value = 0.041). There was a negative interaction effect on an additive scale between MEOHP and BMI on recurrent breast cancer risk (RERI_HR_ =  − 1.97).

**Table 3:** The interaction between MEOHP and hormone receptors as well as BMI on recurrent breast cancer in prospective follow-up (figure as shown in Supplementary Fig. S1).

Metabolite^a^	Prospective patient cohort follow-up (45 new recurrence/544 non-recurrence)				*p*-value for interaction
	ER status, n (%)				
MEOHP	ER-negative	aHR (95% CI)^b^	ER-positive	aHR (95% CI)^b^	
Quartile 1	5 (33.3)/32 (31.1)	1	11 (36.7)/98 (22.7)	1	0.843
Quartile 2	7 (46.7)/34 (33.0)	1.03 (0.32–3.26)	6 (20.0)/98 (22.7)	0.57 (0.21–1.55)	
Quartile 3	1 (6.7)/27 (26.2)	0.23 (0.03–1.93)	4 (13.3)/120 (27.8)	0.18 (0.05–0.66)**	
Quartile 4	2 (13.3)/10 (9.7)	0.85 (0.16–4.46)	9 (30.0)/116 (26.9)	0.49 (0.20–1.19)	

MEOHP	PR status, n (%)				*p*-value for interaction
	PR-negative	aHR (95% CI)^b^	PR-positive	aHR (95% CI)^b^	
Quartile 1	10 (40.0)/36 (23.1)	1	6 (30.0)/93 (24.9)	1	0.794
Quartile 2	7 (28.0)/43 (27.6)	0.56 (0.21–1.49)	6 (30.0)/88 (23.5)	1.00 (0.32–3.09)	
Quartile 3	2 (8.0)/50 (32.1)	0.14 (0.03–0.63)*	3 (15.0)/95 (25.4)	0.26 (0.05–1.33)	
Quartile 4	6 (24.0)/27 (17.3)	0.49 (0.17–1.38)	5 (25.0)/98 (26.2)	0.56 (0.17–1.85)	

MEOHP	HER2 status, n (%)				*p*-value for interaction
	HER2-negative	aHR (95% CI)^b^	HER2-positive	aHR (95% CI)^b^	
Quartile 1	12 (38.7)/93 (24.4)	1	4 (28.6)/36 (26.5)	1	0.964
Quartile 2	7 (22.6)/94 (24.7)	0.60 (0.23–1.52)	6 (42.9)/32 (23.5)	1.17 (0.33–4.21)	
Quartile 3	5 (16.1)/107 (28.1)	0.24 (0.08–0.76)*	0 (0.0)/33 (24.3)	–	
Quartile 4	7 (22.6)/87 (22.8)	0.46 (0.18–1.19)	4 (28.6)/35 (25.7)	0.55 (0.13–2.34)	

MEOHP	BMI status, n (%)				*p*-value for interaction
	BMI < 25	aHR (95% CI)^b^	BMI ≥ 25	aHR (95% CI)^b^	
Quartile 1	8 (25.8)/86 (23.8)	1	8 (61.5)/45 (25.9)	1	0.042*
Quartile 2	10 (32.3)/83 (22.9)	1.13 (0.45, 2.86)	3 (23.1)/49 (28.2)	0.40 (0.10, 1.51)	
Quartile 3	3 (9.7)/101 (27.9)	0.25 (0.07, 0.93)*	1 (7.7)/44 (25.3)	0.11 (0.01, 0.92)*	
Quartile 4	10 (32.3)/92 (25.4)	0.81 (0.32, 2.07)	1 (7.7)/36 (20.7)	0.09 (0.01, 0.77)*	

*HR* hazard ratio, *CI* confidence interval.

^a^The urinary concentration unit of phthalate metabolites were μg/g creatinine and were categorized into quartiles with the first quartile being defined as the reference group.

^b^HR was calculated by multivariable Cox proportional hazards model and adjusted for marriage and radiotherapy, shown as aHR (95% CI).

*p-values < 0.05, **p-values < 0.01.

## Discussion

This study is the first patient cohort design to explore the interaction between phthalate exposure and hormone receptors as well as BMI on recurrent breast cancer. It is suggested that MEOHP were negatively associated with recurrent breast cancer and these effects were modified by hormone receptors and BMI. MEOHP was associated with the lowest breast cancer recurrence risk in the status of ER-positive, PR-negative, and HER2-negative (molecular subtype of breast cancer: luminal A) and the dose–response was U-shaped. The effects of EDCs have often been observed as an NMDR curve in animal, cell culture, and epidemiological studies^27^. EDCs have different mechanisms due to different doses, including cell and tissue specificity, receptor selectivity, receptor competition, and negative feedback^27,28^. An animal study found that adult mice exposed to BPA resulted in mammary cancer and displayed an NMDR curve^29^. An epidemiological study also found an inverted U-shaped association between diabetes and persistent organic pollutants^30^.

Breast cancer patients with hormone receptor positive (HR+)/HER2− subtype (approximating luminal A subtype) had the best survival pattern of 4-year breast cancer-specific survival^31^. Luminal B, HER2‐enriched, and triple‐negative tumors had higher recurrence risk and bad prognosis compared with luminal A tumors^32,33^. A Poland population-based case–control study reported that exposure to organic solvents was positively associated with breast cancer and might be limited to ER−/PR− cases^34^, but little heterogeneous effect by ER status between most phthalate metabolites and breast cancer was observed^13^, which was similar to our study. Few epidemiological studies are available to explore the association between recurrent breast cancer and molecular subtypes in relation to phthalate exposure. The present study suggests that exposure to MEOHP might be associated with decreasing breast cancer recurrence risk, especially in the status of ER-positive, PR-negative, and HER2-negative.

Phthalates can be added in personal care products to hold color and fix fragrance, and women are exposed to phthalates through usage of such products^35^. DEHP, as a plasticizer to increase flexibility, is present in medical devices and construction material^36,37^. Compared to nonusers, the concentration of MiBP is higher in users of perfume^35,38^ and MEOHP is higher in bottled water^38^. Our previous study found that MEHHP, MECPP, MEOHP, and Σ_4_DEHP were positively associated with increased intron 1 methylation level and gene expression in ADAM33 gene and the phenomenon may be related to decreased breast cancer risk^39^. Two studies and a multiethnic cohort study in whites observed an non-significant negative association between MEOHP and breast cancer^13,14,40^; a prospective study and an Alaska study showed that MEOHP were not associated with breast cancer^12,15^. A cross-sectional study in the National Health and Nutrition Examination Survey (NHANES) also found that phthalate metabolites were not significantly associated with breast cancer^41^, but MEOHP was related to male-specific thyroid cancer^16^, and MEHHP, MEOHP, MECPP, MCMHP, MBzP, MiBP, and MnBP were also associated with prostate cancer in a waist ≥ 90 cm subgroup^17^; however, four studies observed that MBzP was negatively associated with breast cancer risk^12–14,40^. So far, the inconsistent results of these studies were still unclear, and this could explain the different results from study designs, races, populations, cultures, environmental, dietary habits, and even different diseases.

The mechanisms of phthalates are nothing more than peroxisome proliferator-activated receptors (PPARs), DNA methylation, and DMA damage. PPARs have three isoforms identified: PPARα, PPARβ/δ, and PPARγ. PPARs play an important role in lipid metabolism and are involved in cellular differentiation and carcinogenesis^42^. PPARγ has significant expression in human mammary adenocarcinomas and reduces the tumor growth of malignant breast epithelial cells^43^. It is associated with longer breast cancer-specific survival and expresses more in luminal ER-positive tumors, suggesting that PPARγ is a better marker for prognosis in luminal breast cancer patients^44^. MEHP and MEOHP bonded to the ligand binding pocket of PPARγ and produced interaction as confirmed by reporter gene assay^45^; therefore, the negative correlation between MEOHP and breast cancer recurrence might be caused by the combination of MEOHP and PPARγ, resulting in reduced risk of breast cancer recurrence in our study. PPARα and PPARγ have opposite effects on human breast cells. PPARα causes the proliferation of breast cells; however, PPARγ inhibits cell proliferation. MEHP activates both human PPARα and PPARγ, but not PPARβ. However, MnBP cannot activate any PPAR isoform, but MnBP is an antagonist against both human PPARβ and PPARγ^46^. Because different phthalates have different mechanisms on different PPARs, this leads to different growth effects on breast cells.

Studies have suggested that the additive scale could better reflect biologic interaction and is important for public health interventions in a prospective study^24,25^. In our study, there was a negative interaction on an additive scale between MEOHP and BMI on recurrent breast cancer. The prevalence of obesity is a global problem and the phthalate exposure, a EDCs, might be one of the reasons for obesity^47,48^. A meta-analysis study indicated that high heterogeneity between studies for MEOHP and obesity was insufficient to perform meta-analysis; however, the association between MEOHP and obesity was positive in most adult and child studies, but there was little statistical significance^49^. The study found adipocyte in culture medium accumulated MEHP^50^ and individuals undergoing weight loss had higher levels of MEP^51^. The negative association between some phthalates and obesity could be explained by the cumulative capacity and lipophilic capacity of phthalates in adipose tissue^52^. A large study also showed that the association between breast cancer-specific mortality and MEOHP was modified by BMI^13^. In our study, whether in BMI < 25 or in BMI ≥ 25, MEOHP was negatively associated with recurrent breast cancer. However, few studies have explored the association between recurrent breast cancer, phthalate exposure, and obesity. For further analysis in the future, the obesity indicators, including BMI, waist circumference, waist-hip ratio, and body fat ratio, should be considered while sample size should be expanded for exploring the association between phthalate exposure, obesity, and recurrent breast cancer.

In sociodemographic characteristics, we found a higher frequency of being single/unmarried in recurrent breast cancer patients. Some studies have found that married breast cancer patients have lower mortality rate than unmarried patients^53,54^. Compared to married women, unmarried women were 1.18-times more likely to have later stage breast cancer at breast cancer diagnosis^55^. Furthermore, married patients were more likely to receive chemotherapy and radiotherapy than those unmarried^53,54^. Two epidemiological studies on pregnant women also found that the concentrations of certain phthalates were higher in unmarried Caucasian women relative to those married^56^. Being unmarried might also be a risk factor for recurrent breast cancer due to lack of care and companionship, and having some undesirable living habits; therefore, further study is needed to explore the relationship between recurrent breast cancer and the sources of phthalate exposure, such as environmental exposure and the use of phthalate products.

Our study has some limitations. Firstly, only a single-spot morning urine was used to evaluate phthalate exposures; however, there were no differences between two consecutive first-morning urine tests, suggesting that the phthalate exposure patterns of women were relatively stable^57^. In addition, phthalates have short biological half-lives and metabolize quickly within 24 h^58^. Previous studies have indicated that the collection of multiple spot urine samples was more conducive to assess most complete phthalate exposures compared with a single urine measurement^59^. The within-person reproducibility of most phthalate metabolites was modest, so the results would tend to be attenuated^60^. In our prospective patient cohort design, due to the non-differential misclassification of phthalate exposure, the association between phthalate exposure and recurrent breast cancer may be underestimated. Secondly, the questionnaire did not include environmental exposure issues, including the use of plastic containers and personal care products. However, the concentrations of phthalate metabolites in urine already represented the phthalate exposures of the body from various sources. Thirdly, since only one-time measurement of phthalate exposures and disease outcome was performed, it was difficult to explore the association between phthalate exposure and recurrent breast cancer; however, in our patient cohort study, we excluded patients who already had recurrence and followed up new patients to recurrence status. Fourthly, although the number of new recurrent samples was small, it could explore the temporary association between the risk of breast cancer recurrence and phthalate exposure in this patient cohort design.

Our study is the first pioneer patient cohort study to assess the interaction between phthalate exposure and hormone receptors as well as BMI on recurrent breast cancer. We found that the negative association between MEOHP and breast cancer recurrence was modified by the hormone receptors and BMI. There was also a negative interaction between MEOHP and BMI on recurrent breast cancer. From a future perspective, it is necessary to recruit large sample sizes and new recurrent breast cancer patients to understand how phthalate exposure could affect the recurrence and prognosis, while the biological mechanism between phthalate exposure and recurrent breast cancer also warrants exploration.

## Supplementary Information

Supplementary Information.

## Data Availability

All data generated or analysed during this study are included in this published article (and its Supplementary Information files).

## Abbreviations

IARC: International Agency for Research on Cancer
MOHW: Ministry of Health and Welfare
EDCs: Endocrine-disrupting chemicals
BMI: Body mass index
KMU: Kaohsiung Medical University
IRB: Institutional Review Board
WHO: World Health Organization
IHC: Immunohistochemistry
ER: Estrogen receptor
PR: Progesterone receptor
HER2: Human epidermal growth factor receptor 2
TNM stage: TNM classification of malignant tumors
MEP: Mono-ethyl phthalate
MnBP: Mono-n-butyl phthalate
MiBP: Mono-isobutyl phthalate
MEHP: Mono-2-ethylhexyl phthalate
MEHHP: Mono-(2-ethyl-5-hydroxyhexyl) phthalate
MECPP: Mono-(2-ethyl-5-carboxypentyl) phthalate
MEOHP: Mono-(2-ethyl-5-oxohexyl) phthalate
MMP: Mono-methyl phthalate
MBzP: Mono-benzyl phthalate
MCPP: Mono-(3-carboxypropyl) phthalate
ESI: Electrospray ionization
LC–MS/MS: Liquid chromatography–tandem mass spectrometry
LOD: Limit of detection
CV: Coefficient of variation
HRs: Hazard ratios
CIs: Confidence intervals
RERI: Relative excess risk due to interaction
NMDR: Non-monotonic dose–response
NHANES: National Health and Nutrition Examination Survey
PPARs: Peroxisome proliferator-activated receptors

## References

[1] Wild CP, Weiderpass E, Stewart BW (2020). World Cancer Report: Cancer Research for Cancer Prevention.

[2] Sung H (2021). Global cancer statistics 2020: GLOBOCAN estimates of incidence and mortality worldwide for 36 cancers in 185 countries. CA Cancer J. Clin..

[3] Dent R (2014). Factors associated with breast cancer mortality after local recurrence. Curr. Oncol..

[4] Sirohi B, Leary A, Johnston SR (2009). Ipsilateral breast tumor recurrence: Is there any evidence for benefit of further systemic therapy?. Breast J..

[5] Centers for Disease Control and Prevention (2019). Fourth Report on Human Exposure to Environmental Chemicals, Updated Tables, January 2019.

[6] Sicinska P (2018). Di-n-butyl phthalate, butylbenzyl phthalate and their metabolites induce haemolysis and eryptosis in human erythrocytes. Chemosphere.

[7] Wallner P, Kundi M, Hohenblum P, Scharf S, Hutter HP (2016). Phthalate metabolites, consumer habits and health effects. Int. J. Environ. Res. Public Health..

[8] Benjamin S (2017). Phthalates impact human health: Epidemiological evidences and plausible mechanism of action. J. Hazard Mater..

[9] Key TJ, Verkasalo PK, Banks E (2001). Epidemiology of breast cancer. Lancet Oncol..

[10] Zuccarello P (2018). Implication of dietary phthalates in breast cancer. A systematic review. Food Chem. Toxicol..

[11] Sheikh IA (2016). Endocrine disruption: In silico perspectives of interactions of di-(2-ethylhexyl)phthalate and its five major metabolites with progesterone receptor. BMC Struct. Biol..

[12] Reeves KW (2019). Urinary phthalate biomarker concentrations and postmenopausal breast cancer risk. J. Natl. Cancer Inst..

[13] Parada H (2018). Urinary phthalate metabolite concentrations and breast cancer incidence and survival following breast cancer: The Long Island Breast Cancer Study Project. Environ. Health Perspect..

[14] Lopez-Carrillo L (2010). Exposure to phthalates and breast cancer risk in northern Mexico. Environ. Health Perspect..

[15] Holmes AK (2014). Case-control study of breast cancer and exposure to synthetic environmental chemicals among Alaska Native women. Int. J. Circumpolar Health.

[16] Liu C (2020). Urinary biomarkers of phthalates exposure and risks of thyroid cancer and benign nodule. J. Hazard Mater.

[17] Chuang SC (2020). Phthalate exposure and prostate cancer in a population-based nested case-control study. Environ. Res..

[18] Silva MJ (2003). Glucuronidation patterns of common urinary and serum monoester phthalate metabolites. Arch. Toxicol..

[19] Hines EP, Calafat AM, Silva MJ, Mendola P, Fenton SE (2009). Concentrations of phthalate metabolites in milk, urine, saliva, and Serum of lactating North Carolina women. Environ. Health Perspect..

[20] Calafat AM (2015). Optimal exposure biomarkers for nonpersistent chemicals in environmental epidemiology. Environ. Health Perspect..

[21] Engel SM, Wolff MS (2013). Causal inference considerations for endocrine disruptor research in children's health. Annu. Rev. Public Health.

[22] Yaghjyan L, Sites S, Ruan Y, Chang SH (2015). Associations of urinary phthalates with body mass index, waist circumference and serum lipids among females: National Health and Nutrition Examination Survey 1999–2004. Int. J. Obes. (Lond.).

[23] Starling AP (2015). Predictors and long-term reproducibility of urinary phthalate metabolites in middle-aged men and women living in urban Shanghai. Environ. Int..

[24] Knol MJ, van der Tweel I, Grobbee DE, Numans ME, Geerlings MI (2007). Estimating interaction on an additive scale between continuous determinants in a logistic regression model. Int. J. Epidemiol..

[25] Rod NH, Lange T, Andersen I, Marott JL, Diderichsen F (2012). Additive interaction in survival analysis: Use of the additive hazards model. Epidemiology.

[26] VanderWeele TJ (2011). Causal interactions in the proportional hazards model. Epidemiology.

[27] Vandenberg LN (2012). Hormones and endocrine-disrupting chemicals: Low-dose effects and nonmonotonic dose responses. Endocr. Rev..

[28] Dutta S, Haggerty DK, Rappolee DA, Ruden DM (2020). Phthalate exposure and long-term epigenomic consequences: A review. Front. Genet..

[29] Jenkins S, Wang J, Eltoum I, Desmond R, Lamartiniere CA (2011). Chronic oral exposure to bisphenol A results in a nonmonotonic dose response in mammary carcinogenesis and metastasis in MMTV-erbB2 mice. Environ. Health Perspect..

[30] Lee DH (2010). Low dose of some persistent organic pollutants predicts type 2 diabetes: A nested case-control study. Environ. Health Perspect..

[31] Howlader N, Cronin KA, Kurian AW, Andridge R (2018). Differences in breast cancer survival by molecular subtypes in the United States. Cancer Epidemiol. Biomark. Prev..

[32] Gao JJ, Swain SM (2018). Luminal A breast cancer and molecular assays: A review. Oncologist.

[33] Chen J (2014). The efficacy of molecular subtyping in predicting postoperative recurrence in breast-conserving therapy: A 15-study meta-analysis. World J. Surg. Oncol..

[34] Peplonska B (2010). Occupational exposure to organic solvents and breast cancer in women. Occup. Environ. Med..

[35] Parlett LE, Calafat AM, Swan SH (2013). Women's exposure to phthalates in relation to use of personal care products. J. Expo Sci. Environ. Epidemiol..

[36] Schettler T (2006). Human exposure to phthalates via consumer products. Int. J. Androl..

[37] Sampson J, De Korte D (2011). DEHP-plasticised PVC: Relevance to blood services. Transfus. Med..

[38] Romero-Franco M (2011). Personal care product use and urinary levels of phthalate metabolites in Mexican women. Environ. Int..

[39] Yang PJ (2018). Breast cancer is associated with methylation and expression of the a disintegrin and metalloproteinase domain 33 (ADAM33) gene affected by endocrinedisrupting chemicals. Oncol. Rep..

[40] Wu AH (2021). Urinary phthalate exposures and risk of breast cancer: The Multiethnic Cohort study. Breast Cancer Res..

[41] Morgan M, Deoraj A, Felty Q, Roy D (2017). Environmental estrogen-like endocrine disrupting chemicals and breast cancer. Mol. Cell Endocrinol..

[42] Berger J, Moller DE (2002). The mechanisms of action of PPARs. Annu. Rev. Med..

[43] Mueller E (1998). Terminal differentiation of human breast cancer through PPAR gamma. Mol. Cell.

[44] Abduljabbar R (2015). Prognostic and biological significance of peroxisome proliferator-activated receptor-gamma in luminal breast cancer. Breast Cancer Res. Treat..

[45] Kratochvil I (2019). Mono(2-ethylhexyl) phthalate (MEHP) and mono(2-ethyl-5-oxohexyl) phthalate (MEOHP) but not di(2-ethylhexyl) phthalate (DEHP) bind productively to the peroxisome proliferator-activated receptor gamma. Rapid Commun. Mass Spectrom..

[46] Venkata NG (2006). Mono(2-ethylhexyl)phthalate and mono-n-butyl phthalate activation of peroxisome proliferator activated-receptors alpha and gamma in breast. Toxicol. Lett..

[47] Hruby A, Hu FB (2015). The epidemiology of obesity: A big picture. Pharmacoeconomics.

[48] Baillie-Hamilton PF (2002). Chemical toxins: A hypothesis to explain the global obesity epidemic. J. Altern. Complement Med..

[49] Ribeiro C (2019). Association between the exposure to phthalates and adiposity: A meta-analysis in children and adults. Environ. Res..

[50] Chiang HC (2016). Mono(2-ethylhexyl)phthalate accumulation disturbs energy metabolism of fat cells. Arch. Toxicol..

[51] Dirtu AC (2013). Phthalate metabolites in obese individuals undergoing weight loss: Urinary levels and estimation of the phthalates daily intake. Environ. Int..

[52] Jackson E, Shoemaker R, Larian N, Cassis L (2017). Adipose tissue as a site of toxin accumulation. Compr. Physiol..

[53] Liu YL (2019). Marital status is an independent prognostic factor in inflammatory breast cancer patients: An analysis of the surveillance, epidemiology, and end results database. Breast Cancer Res. Treat..

[54] Martinez ME (2017). Prognostic significance of marital status in breast cancer survival: A population-based study. PLoS ONE.

[55] Hinyard L, Wirth LS, Clancy JM, Schwartz T (2017). The effect of marital status on breast cancer-related outcomes in women under 65: A SEER database analysis. Breast.

[56] Wenzel AG (2018). Prevalence and predictors of phthalate exposure in pregnant women in Charleston, SC. Chemosphere.

[57] Hoppin JA, Brock JW, Davis BJ, Baird DD (2002). Reproducibility of urinary phthalate metabolites in first morning urine samples. Environ. Health Perspect..

[58] Agency for Toxic Substances and Disease Registry (ATSDR) (2019). Toxicological Profile for Di(2-ethylhexyl)phthalate (DEHP).

[59] Preau JL, Wong LY, Silva MJ, Needham LL, Calafat AM (2010). Variability over 1 week in the urinary concentrations of metabolites of diethyl phthalate and di(2-ethylhexyl) phthalate among eight adults: An observational study. Environ. Health Perspect..

[60] Townsend MK, Franke AA, Li X, Hu FB, Eliassen AH (2013). Within-person reproducibility of urinary bisphenol A and phthalate metabolites over a 1 to 3 year period among women in the Nurses’ Health Studies: A prospective cohort study. Environ. Health.

