# Supplementary material for: Association between recurrent breast cancer and phthalate exposure modified by hormone receptors and body mass index
Source: Sci Rep. 2022 Feb 21;12:2858. doi: 10.1038/s41598-022-06709-3 (PMC8861041; doi:10.1038/s41598-022-06709-3)
Supplement: Supplementary file 1 — Supplementary Information. [file 41598_2022_6709_MOESM1_ESM.pdf]

Supplementary information

Association between recurrent breast cancer and phthalate exposure modified by hormone receptors and body mass index

Pei-Jing Yang, Ming-Feng Hou, Fu Ou-Yang, Tsung-Hua Hsieh, Yen-Jung Lee, Eing-Mei Tsai*, and Tsu-Nai Wang*

Contents of Figure and Table

Table S1. Urinary phthalate metabolites measured by LC-MS/MS.

Table S2. Spearman correlation coefficients (ρ) for the urinary phthalate metabolite concentrations (n = 636).

Table S3. The differences of urinary phthalate metabolite concentrations between new recurrent and non-recurrent breast cancer patients.

Fig. S1. The interaction between MEOHP and hormone receptors as well as BMI on recurrent breast cancer in prospective follow-up.
The urinary concentration unit of phthalate metabolites were μg/g creatinine and were categorized into quartiles with the first quartile being defined as the reference group. The black squares represent the adjusted hazard ratio (aHR) for each quartile and the horizontal lines represent the 95% confidence interval (CI); while the vertical dotted line represents a hazard ratio of 1.0. Note: Q, quartile. *P-values < 0.05, **P-values < 0.01.

Table S1. Urinary phthalate metabolites measured by LC-MS/MS.

Phthalate	Metabolite	Molar Mass	LOD (μg/L)	Detection rate (%)	Precursor/ Product ions (*m*/z)	Isotope-labeled precursor/ Product ions (*m*/z)	Retention time (min)	Collision energy (V)	Tube lens (V)
**DEP**	MEP	194	5	98.4%	193/77	197/81	3.39	13	30
**DnBP**	MnBP	222	5	100.0%	221/77	225/81	8.35	20	10
**DiBP**	MiBP	222	5	99.5%	221/77	225/81	7.97	20	10
**DEHP**	MEHP	278	0.5	100.0%	277/134	281/138	14.02	19	22
	MEHHP	294	0.5	99.8%	293/121	297/125	7.42	21	21
	MECPP	308	0.5	99.8%	307/159	311/164	7.72	13	7
	MEOHP	292	0.5	100.0%	291/121	295/124	8.37	21	9
**DMP**	MMP	180	5	0%	179/77	183/81	2.59	19	10
**BBzP**	MBzP	256	1	68.9%	255/183	259/187	10.00	15	4

Note: LOD, limit of detection; DEP, diethyl phthalate; DnBP, di-n-butyl phthalate; DiBP, di-isobutyl phthalate; DEHP, di-2-ethylhexyl phthalate; DMP, dimethyl phthalate; BBzP, butyl benzyl phthalate; MEP, mono-ethyl phthalate; MnBP, mono-n-butyl phthalate; MiBP, mono-isobutyl phthalate; MEHP, mono-2-ethylhexyl phthalate; MEHHP, mono-(2-ethyl-5-hydroxyhexyl) phthalate; MECPP, mono-(2-ethyl-5-carboxypentyl) phthalate; MEOHP, mono-(2-ethyl-5-oxohexyl) phthalate; MMP, mono-methyl phthalate; MBzP, mono-benzyl phthalate.

Table S2. Spearman correlation coefficients (ρ) for the urinary phthalate metabolite concentrations (n = 636).

**Metabolites**	**MEP**	**MnBP**	**MiBP**	**MEHP**	**MEHHP**	**MECPP**	**MEOHP**	**Σ_4DEHP**
**MEP**	1	0.292**	0.300**	0.213**	0.130**	0.209**	0.207**	0.221**
**MnBP**		1	0.694**	0.537**	0.337**	0.429**	0.517**	0.533**
**MiBP**			1	0.644**	0.186**	0.423**	0.465**	0.527**
**MEHP**				1	0.324**	0.577**	0.652**	0.776**
**MEHHP**					1	0.693**	0.820**	0.738**
**MECPP**						1	0.814**	0.901**
**MEOHP**							1	0.920**
**Σ_4DEHP**								1

Note: Σ_4DEHP, the sum of MEHP, MEHHP, MECPP, and MEOHP urinary phthalate metabolite concentrations. ***P*-values < 0.01.

Table S3. The differences of urinary phthalate metabolite concentrations between new recurrent and non-recurrent breast cancer patients.

Metabolites (μg/g)[a]	Prospective follow-up		*P*-values[b]	*P*-values[c]
	New recurrence (n = 45)	Non-recurrence (n = 544)		
**MEP**	21.3 (14.2, 32.0)	23.9 (21.5, 26.7)	0.556	0.578
**MnBP**	18.7 (14.0, 24.9)	20.1 (18.5, 21.8)	0.624	0.463
**MiBP**	5.2 (3.7, 7.2)	7.3 (6.5, 8.2)	0.094	0.167
**MEHP**	5.7 (4.2, 7.7)	7.7 (6.9, 8.6)	0.124	0.115
**MEHHP**	11.3 (8.5, 15.0)	12.7 (11.7, 13.7)	0.421	0.592
**MECPP**	21.3 (16.2, 28.0)	22.8 (21.0, 24.7)	0.660	0.575
**MEOHP**	6.9 (5.4, 8.8)	8.2 (7.6, 8.9)	0.211	0.102
**Σ_4DEHP**	49.7 (38.8, 63.7)	57.5 (53.3, 62.0)	0.293	0.209

NOTE: Σ_4DEHP, the sum of MEHP, MEHHP, MECPP, and MEOHP urinary phthalate metabolite concentrations; CI, confidence interval. [a]The concentration unit of metabolites was μg/g creatinine and expressed as the geometric means (95% CI). [b]The metabolite concentrations were ln-transformed for Independent sample *t*-test. [c]*P*-values were calculated for continuous variables by Mann-Whitney U test.

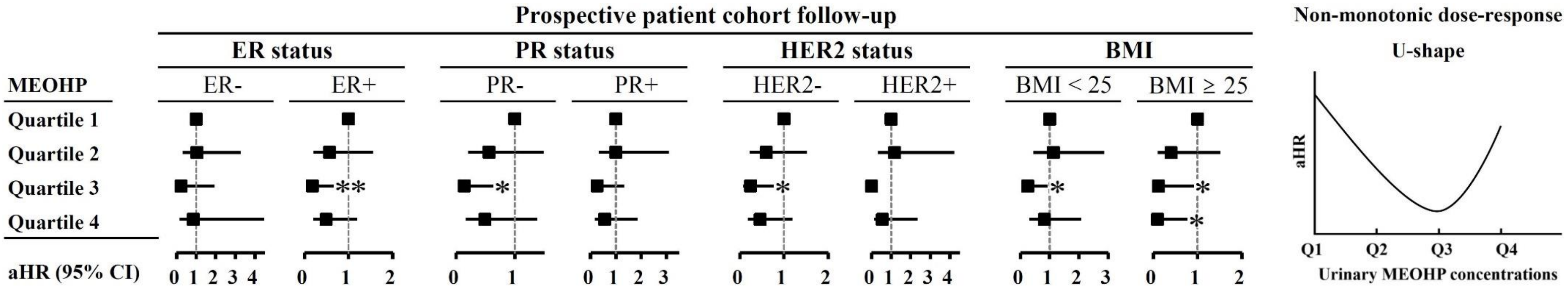
Prospective patient cohort follow-up
ER status
PR status
HER2 status
BMI
MEOHP
ER-
ER+
PR-
PR+
HER2-
HER2+
BMI < 25
BMI ≥ 25
Quartile 1
Quartile 2
Quartile 3
Quartile 4
aHR (95% CI)
0 1 2 3 4
0 1 2
0 1
0 1 2 3
0 1 2
0 1 2 3 4
0 1 2 3
0 1 2
Non-monotonic dose-response
U-shape
aHR
Q1 Q2 Q3 Q4
Urinary MEOHP concentrations
